# Angiotensin Type-1 Receptor Inhibition Reduces NLRP3 Inflammasome Upregulation Induced by Aging and Neurodegeneration in the *Substantia Nigra* of Male Rodents and Primary Mesencephalic Cultures

**DOI:** 10.3390/antiox11020329

**Published:** 2022-02-08

**Authors:** Aloia Quijano, Carmen Diaz-Ruiz, Andrea Lopez-Lopez, Begoña Villar-Cheda, Ana Muñoz, Ana I. Rodriguez-Perez, Jose L. Labandeira-Garcia

**Affiliations:** 1 Laboratory of Cellular and Molecular Neurobiology of Parkinson’s Disease, Research Center for Molecular Medicine and Chronic Diseases (CIMUS), Department of Morphological Sciences, IDIS, University of Santiago de Compostela, 15782 Santiago de Compostela, Spain; aloia.quijano.ocampo@usc.es (A.Q.); mdelcarmen.diaz@usc.es (C.D.-R.); andrealopez.lopez@usc.es (A.L.-L.); bego.villar@usc.es (B.V.-C.); anamaria.munoz@usc.es (A.M.); anai.rodriguez@usc.es (A.I.R.-P.); 2 Networking Research Center on Neurodegenerative Diseases (CiberNed), 28031 Madrid, Spain

**Keywords:** aged, dopamine, inflammation, inflammaging, neuroinflammation, oxidative stress, Parkinson’s disease, renin–angiotensin system

## Abstract

The tissue renin–angiotensin system (RAS) has been shown to be involved in prooxidative and proinflammatory changes observed in aging and aging-related diseases such as dopaminergic degeneration in Parkinson’s disease (PD). We studied the activation of the NLRP3 inflammasome in the *substantia nigra* with aging and early stages of dopaminergic degeneration in PD models and, particularly, if the brain RAS, via its prooxidative proinflammatory angiotensin II (AngII) type 1 (AT1) receptors, mediates the inflammasome activation. *Nigras* from aged rats and mice and 6-hydroxydopamine PD models showed upregulation in transcription of inflammasome-related components (NLRP3, pro-IL1β and pro-IL18) and IL1β and IL18 protein levels, which was inhibited by the AT1 receptor antagonist candesartan. The role of the AngII/AT1 axis in inflammasome activation was further confirmed in rats intraventricularly injected with AngII, and in primary mesencephalic cultures treated with 6-hydroxydopamine, which showed inflammasome activation that was blocked by candesartan. Observations in the *nigra* of young and aged AT1 and AT2 knockout mice confirmed the major role of AT1 receptors in nigral inflammasome activation. In conclusion, the inflammasome is upregulated by aging and dopaminergic degeneration in the *substantia nigra*, possibly related with a decrease in dopamine levels, and it is mediated by the AngII/AT1 axis.

## 1. Introduction

A proinflammatory state is observed in most tissues during aging (inflammaging), and facilitates development of aging-related chronic diseases [1,2]. In the brain, aging-related neuroinflammation promotes progression of major neurodegenerative diseases such as Parkinson’s disease (PD) and Alzheimer’s disease [3]. The renin–angiotensin system (RAS), particularly local or tissue RAS, has been related to prooxidative and proinflammatory changes observed in aging and aging-related diseases in several peripheral tissues, particularly cardiovascular and renal tissues [4]. The brain has a local RAS, and dysregulation of brain RAS has been observed in aged brains [5,6] and animal models of neurodegenerative diseases, including PD [7,8]. 

The tissue RAS physiological function involves the correct balance between two arms, which counteract each other: a prooxidative and proinflammatory axis mainly constituted by angiotensin II (AngII) and angiotensin type 1 (AT1) receptors, and an anti-oxidative and anti-inflammatory axis consisting of AngII/AT2 receptors and Ang1-7/Mas receptors (MasR) [9,10]. The *substantia nigra* of aged animals and PD models shows hyperactivation of the AngII/AT1 prooxidative axis, which leads to increase in several markers of oxidative stress (OS) and neuroinflammation [8,11]. A major mechanism involved in this effect is the AngII/AT1-induced activation of the NADPH–oxidase (NOX) complex leading to superoxide production in neurons and microglia [12,13], which interacts with mitochondria leading to further production of OS [14,15], and triggers microglial activation and neuroinflammation. However, the mechanisms that play a role in promoting the angiotensin-related neuroinflammatory response in aging and PD have not been clarified. 

The inflammasome is a multiprotein complex that mediates inflammation in response to both pathogens and inflammatory triggers and can be divided into subtypes based on the different combinations of molecules. The most well-known inflammasome is the leucine-rich-repeat- and pyrin-domain-containing 3 (NLRP3) inflammasome [16]. The inflammasomes have been particularly studied in myeloid lineages, where inflammasome activation leads to caspase 1-dependent activation and production of interleukin (IL) 1β and IL18 [17]. In the brain, inflammasome proteins have been observed to play a major role in the development of neuroimmune, and neurodegenerative diseases [18]. In particular, several recent studies have shown that NLRP3 inflammasome is upregulated in PD and PD models [19,20,21]. Brain NLRP3 is predominantly located in microglia [22,23], although some studies have observed that astrocytes [24] and dopaminergic neurons [25] may also express the NLRP3 inflammasome.

The activation of the NLRP3 inflammasome complex requires two major steps [26]. First, in the priming step (step 1), a proinflammatory stimulus, such as a damage-associated molecular pattern or a pathogen-associated molecular pattern binds a pattern recognition receptor. This interaction activates the NF-κβ-pathway. NF-κβ then translocates into the nucleus, increasing the transcription of the NLRP3 inflammasome-related components, such as the NLRP3, pro-IL1β and pro-IL18. Second, in the activation step (step 2), a second stimulus triggers the NLRP3 inflammasome complex assembly. This induces the transformation of the procaspase-1 to caspase-1, catalyzing the production of IL1β and IL18 from pro-IL1β and pro-IL18, which promote inflammation [27,28]. In the present work, we studied if there is an activation of the NLRP3 inflammasome in the aged *substantia nigra* and early stages of dopaminergic degeneration in PD models, and if the brain RAS, via its prooxidative proinflammatory AT1 receptors mediates the inflammasome activation.

## 2. Materials and Methods

### 2.1. Experimental Design

Possible activation of NLRP3 inflammasome in the aged *substantia nigra* was studied in aged rats and aged WT mice. Activation of NLRP3 inflammasome in early stages of dopaminergic degeneration was studied in the *substantia nigra* of young adult rats 10 days after intrastriatal injection of the neurotoxin 6-hydroxydopamine (6-OHDA), and in primary (neuron-glia) cultures of ventral mesencephalon treated with 6-OHDA for 8 h. At 10 days post 6-OHDA intrastriatal injection, most of the affected nigral dopaminergic neurons are still under the degenerative process, and there are high levels of neuroinflammatory response in the *substantia nigra* [29]. To study the possible role of the prooxidative proinflammatory arm of the brain RAS in activation of NLRP3 inflammasome in the *substantia nigra* of aged rodents and PD models, rats and cultures were treated with the AT1 receptor antagonist candesartan, and AT1 and AT2 knockout (KO) mice were also analyzed. In addition, rats were treated with AngII in the presence or absence of candesartan. Since AngII is not able to cross a normal blood–brain barrier, AngII was injected in the III ventricle. Activation of the NLRP3 inflammasome was analyzed by determination of transcription of inflammasome-related components (NLRP3, pro-IL1β and pro-IL18; step 1), using quantitative RT-PCR (qRT-PCR), and by determination of IL1β and IL18 protein levels (step 2) using Western blot (WB).

### 2.2. Young Adult and Aged Rats and Mice

Young adult (10-week-old) and aged (18- to 20-month-old) male Sprague Dawley rats, and young adult (10-week-old) and aged (18- to 20-month-old) mice were used. Mice were young (*n* = 12) and aged (*n* = 10) wild-type (WT, C57BL-6 mice from Charles River, L’Arbresle, France), and KO male mice. KO mice were young (*n* = 6) and aged (*n* = 6) homozygous AT2 deficient mice (AT2 KO, gift of Dr. Daniel Henrion) and homozygous AT1a deficient mice (*n* = 6 young, *n* = 6 aged; AT1 KO; AT1a is the major mouse AT1 isoform and the closest murine homolog to the single human AT1; Jackson Laboratory, Bar Harbor, ME, USA). In the present study, we decided on the use of male animals because we have shown in several previous studies that females have a marked downregulation of AT1 and upregulation of AT2 receptors [30,31]. Therefore, any possible role of AT1 in NLRP3 activation would be more easily detected in males and minimized in females. 

Animals were housed under a 12 h light/dark cycle and at a constant temperature (RT; 21–22 °C). Rats and mice were fed *ad libitum* with a convenient pelleted complete diet (SAFE, A04). The animal experiments were performed according to Directives 2010/63/EU and 86/609/CEE. All the protocols involving the use of animals were previously authorized by the corresponding committee of the University of Santiago de Compostela. For surgical experiments, rats and mice were anaesthetized with ketamine/xylazine (Richter Pharma). Some rats from each group were treated with the AT1 receptor antagonist candesartan cilexetil (see below). Immediately after, rats or mice were killed, and the brains were removed from the skull and placed in a rat or mouse stainless-steel brain matrix (51388, Stoelting Co., Wood Dale, IL, USA). Coronal Brain slices were obtained with a tissue chopper. The ventral mesencephalon was dissected on a pre-cooled glass plate, and quickly frozen at −80 °C and stored. Tissue samples were processed for RNA and protein analysis by qRT-PCR and WB.

### 2.3. In Vivo Induction of Partial Dopaminergic Degeneration with the Dopaminergic Neurotoxin 6-Hydroxydopamine (6-OHDA)

For *in vivo* models of partial and early stages of dopaminergic degeneration, young male rats were split into three groups. A first group of animals (*n* = 6) were unilaterally injected in the right striatum with 6-OHDA (H4381, Sigma, St. Louis, MO, USA); 10 µg/3.5 µL of saline containing 0.2% ascorbic acid (A4544, Sigma). Stereotaxic coordinates were 1.0 mm anterior to bregma, 3.0 mm right of midline, and 5.5 mm ventral to the dura; tooth bar at −3.3. The neurotoxin was injected with a 10 μL syringe (Hamilton, Bonaduz, Switzerland), which was handled with a motorized injector (Stoelting; rate of 0.5 µL/min). After injection, the needle was left *in situ* for 5 min. To prevent uptake of 6-OHDA by noradrenergic terminals, rats were treated with the selective inhibitor for the norepinephrine transporter desipramine (25 mg/kg i.p., Sigma) for 30 min prior to intrastriatal injection with 6-OHDA or vehicle. A second group of sham control rats (*n* = 6) was injected with 3.5 μL of 0.2% ascorbic acid in sterile saline in the right the striatum as for the previous group and using different syringes. A third group of rats (*n* = 6) was injected with 6-OHDA, as described above, but rats received treatment with candesartan cilexetil, as detailed below, from 2 weeks before the 6-OHDA injection until sacrifice. Finally, rats were killed 10 days post injection and processed as above to study the early effects of the dopaminergic lesion and neuroinflammatory response. Please note that the effects (i.e., dopaminergic degeneration) of intrastriatal 6-OHDA lesion are complete or almost complete around 3 weeks after lesion [29,32].

### 2.4. Treatment of Rats with Candesartan

As indicated above, some of the rats were treated with the AT1 receptor antagonist candesartan. Candesartan is a specific AT1 blocker that can cross the blood–brain barrier. A low dose of candesartan does not have significant effect on blood pressure and inhibits AngII effects in the central nervous system (CNS) [6,33,34]. For the experiments in which we compared young adult and aged rats, animals received candesartan cilexetil (1 mg/kg/day; AstraZeneca) or vehicle for two weeks (14 successive days). For experiments in which rats were lesioned (to obtain dopaminergic neuron degeneration), animals were treated for 2 weeks prior to surgeries, plus 10 days after injections, until sacrifice. The powered candesartan was administered orally mixed with “Nocilla” hazelnut-cream (Nutrexpa, Barcelona, Spain).

### 2.5. In Vitro Induction of Dopaminergic Degeneration. Primary Mesencephalic Neuron-Glia Cultures

Ventral mesencephalon was extracted from developing brains of rat E14 embryos (i.e., 14 gestation days). The tissue was disrupted first by enzymatic digestion with 0.1% trypsin (Sigma) and 0.05% DNase (Sigma) dissolved in DMEM (Invitrogen Life Technologies, Paisley, Scotland, UK) for 20 min at 37 °C. Afterwards, cells were washed with DNase/DMEM and mechanically disrupted. The cell suspension was subjected to centrifugation (50× *g* for 5 min). After that, the supernatant was carefully removed. Finally, the cells were resuspended in DMEM/HAMS F12 (1:1) medium to the final volume required for 12-well plates (Falcon, Becton Dickinson, Franklin Lakes, NJ, USA) at a density of 1.5 × 10^5^ cells/cm^2^ (plates had been previously coated with poly-L-lysine, 100 μg/mL; Sigma). The DMEM/HAMS F12 (1:1) medium was supplemented with 10% fetal bovine serum (FBS; BiochromKG, Berlin, Germany)]. Cells were cultured for seven days in a humidified incubator with the following conditions: 37 °C, 5% CO_2_, 95% air. After 3 days, the medium was replaced with fresh culture medium. At day 7, culture medium was eliminated and replaced with non-supplemented media. Culture plates were divided into 3 groups: (1) control plates (*n* = 6); (2) plates treated with 6-OHDA (*n* = 6; 10 µM in sterile saline containing 0.2% ascorbic acid; Sigma); (3) plates pretreated with the AT1 receptor blocker candesartan (*n* = 6; 10 µM, #4791, Tocris) for 30 min before treatment with 6-OHDA. After 8 h, cells were washed and processed for qRT-PCR and WB. 

### 2.6. Administration of Intraventricular AngII in Rats

Young (3–4-month-old) male rats were divided into three groups. (1) Sham control rats (*n* = 6) were injected with 3 μL of sterile saline in the third ventricle. A 10 µL Hamilton syringe handled with a motorized injector (Stoelting) was used to inject the solution at a rate of 0.5 µL/min. Stereotaxic coordinates were 0.8 mm posterior to bregma, midline, 6.5 mm ventral to the dura, and tooth bar at (0), based on previous results [35]. (2) A second group of rats (*n* = 6) was injected as above with AngII (Sigma; 5 µg/3 µL sterile saline). (3) A third group of rats (*n* = 6) was treated with the AT1 receptor antagonist candesartan (Astra Zeneca; oral administration; 1 mg/kg/day) for 2 weeks; then, these rats were injected with AngII as above. Rats were killed 24 h after AngII or saline injections and processed for qRT-PCR and WB.

### 2.7. RNA Extraction and qRT-PCR

Homogenates of *substantia nigra* tissue from rat ventral midbrain and primary mesencephalic cultures were extracted with Trizol following the instructions of the manufacturer (Invitrogen, Paisley, UK). One µg of total RNA was reverse-transcribed with nucleoside triphosphates containing deoxyribose, random primers, and Moloney murine leukemia virus (MMLV) reverse transcriptase (200U; Invitrogen) in order to obtain complementary DNA. qRT-PCRs were performed using a QuantStudio3 platform (Applied Biosystems, Foster City, CA, USA). β-Actin was used as housekeeping gene and was amplified in parallel with the genes of interest. To calculate the relative mRNA expression levels, we used the comparative cycle threshold (Ct) values method. The expression of each gene of interest was obtained as relative to the β-Actin housekeeping transcripts. PrimerBLAST software was used to design oligonucleotide primers. Primer sequences were as follows: for rat NLRP3, forward 5′-ACGAAGCAATGCCCTTGGAG-3′, reverse 5′-GGCTGCAGTTGTCTAACTCCA-3′; for rat pro-IL1β, forward 5′-GGCAACTGTCCCTGAACTCA-3′, reverse 5′-TGTCGAGATGCTGCTGTGA-3′; for rat pro-IL18, forward 5′-GCTGCCATACCAGAAGAAGGC-3′, reverse 5′-AGTGGTCTGATTCCAAGTCTCCATT-3′; for rat TH, forward 5′-GGCTTCTCTGACCAGGTGTATCG-3′, reverse 5′-GCAATCTCTTCCGCTGTGTATTCC-3′; for rat IBA1, forward 5′-CAGGAAGAGAGGTTGGATGGGA-3′, reverse 5′-TCGTCTTGAAGGCCTCCAGTT-3′ and for rat β-actin, forward 5′-TCGTGCGTGACATTAAAGAG-3′, reverse 5′-TGCCACAGGATTCCATACC-3′. For mouse, the following primers were used: for NLRP3, forward 5′- ACCCACAACCACAGCCTTCG-3′, reverse 5′- CACCCAACTGTAGGCTCTGC-3′; for IL1β, forward 5′-GCTATGGCAACTGTTCCTGA-3′, reverse 5′-TGATGTGCTGCTGCGAGA-3′ and for IL18, forward 5′-TGGCTGCCATGTCAGAAGACT-3′, reverse 5′-AGTTGTCTGATTCCAGGTCTCCATT-3′.

### 2.8. Western Blot Analysis

Tissue from rat ventral midbrain and primary mesencephalic cultures was lysed in RIPA buffer supplemented with P8340 protease inhibitor cocktail (Sigma) and PMSF (Sigma P7626). Cellular and tissue lysates were sonicated for 3 s and centrifuged at 14,000× *g*. Protein concentrations were measured by the BCA protein assay (Pierce 23225). Thirty µg of the obtained homogenates were treated with Laemmli buffer containing DTT and separated by 14% Bis–Tris polyacrylamide gel, and then transferred onto PVDF membranes (BioRad). The membranes were incubated overnight with hamster anti-IL1β antibody (Santa Cruz sc-12742; 1:100) or rabbit anti-IL18 antibody (Abcam ab191860; 1:1000). Blots were reproved for mouse anti-α-tubulin antibody (Abcam ab185067; 1:50,000), used as a loading control. The HRP-conjugated secondary antibodies used were mouse anti-hamster HRP (Santa Cruz, sc-2789, 1:2500) and mouse anti-rabbit HRP (Santa Cruz, sc-2357, 1:3500) and immunoreactivity was detected with an Immobilon Crescendo Western HRP substrate (Millipore, WBLUR0100) and imaged using a chemiluminescence detection system (Molecular Imager ChemiDoc XRS System, Bio-Rad). Protein expression levels of each sample were determined by densitometry of the corresponding band and expressed relative to the α-tubulin band value. We then normalized the data relative to the mean value obtained for the control group of the same batch to neutralize any variability among batches. 

### 2.9. Statistical Analysis

All data resulted from groups of *n* = 6 animals and are presented using box plots with boxes representing the Interquartile range (IQR) and the median and whiskers representing the maximum value (upper whisker) and the minimum (lower whisker). IQR: Interquartile range.The Student’s test was used for two-group comparisons and one-way ANOVA followed by Student–Newman–Keuls was used for multiple comparisons. Before each student´s and ANOVA test, the normality of populations and homogeneity of variances were tested. Statistical significances were considered at *p* < 0.05. Statistical analyses were performed using GraphPad Prism 8.0.1.244.

## 3. Results

### 3.1. Aging-Related Upregulation of NLRP3 Inflammasome Is Mediated by AT1 Receptors

**Figure 1:**
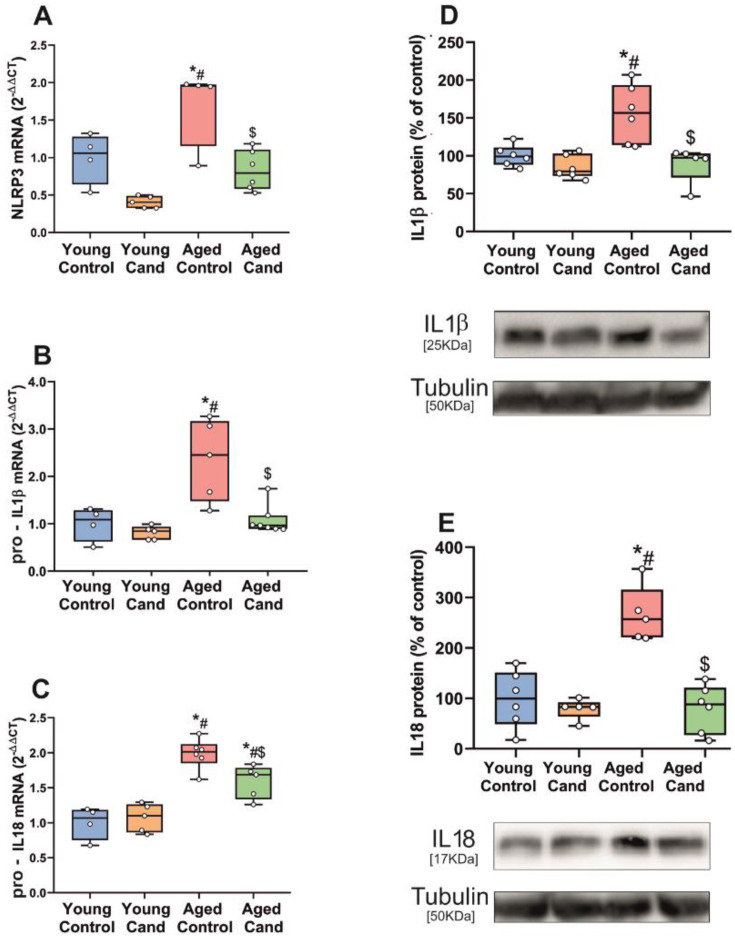
qRT-PCR and WB analysis of NLRP3 inflammasome markers in *substantia nigra* of rats. mRNA expression of NLRP3, pro-IL1β and pro-IL18 inflammasome markers of aged rats significantly increased relative to young rats (**A**–**C**). Treatment of aged rats with the AT1 antagonist candesartan (Cand) significantly inhibited upregulation of the mRNA inflammasome markers (**A**–**C**). Protein expression of IL1β and IL18 significantly increased in aged rats compared to young rats, which was inhibited with candesartan (**D**,**E**). To determine the relative mRNA expression of each gene of interest vs. the β-Actin housekeeping transcripts, we used the comparative Ct values method (2^−ΔΔCt^). Protein expression was determined relative to the α-tubulin band value and normalized to the control values (100%). Data distribution is shown using box plots with boxes representing the Interquartile range (IQR) and the median and whiskers representing the maximum value (upper whisker) and the minimum (lower whisker). * *p* < 0.05 relative to young control rats; # *p* < 0.05 relative to young rats + candesartan, $ *p* < 0.05 relative to aged control rats.

In the *substantia nigra* of aged rats, we observed a significant increase in the expression of mRNA of major NLRP3 inflammasome components (NLRP3, pro-IL1β and pro-IL18) relative to the expression in the *substantia nigra* of young rats. Administration of the AT1 antagonist candesartan led to a marked decrease in mRNA for the NLRP3 components. In young rats, candesartan did not produce significant decreases in mRNA expression of pro-IL1β and pro-IL18 in the *substantia nigra*, and only a significant decrease in the mRNA expression for NLRP3 was detected (Figure 1A–C). Final production of IL1β and IL18 protein was determined by WB. We observed a significant increase in levels of IL1β and IL18 in the *substantia nigra* of aged rats relative to the *substantia nigra* of young rats. As indicated above for step 1 markers, treatment with the AT1 antagonist candesartan significantly reduced the protein levels of IL1β and IL18 in aged rats, but did not significantly affect the levels in young controls (Figure 1D,E).

**Figure 2:**
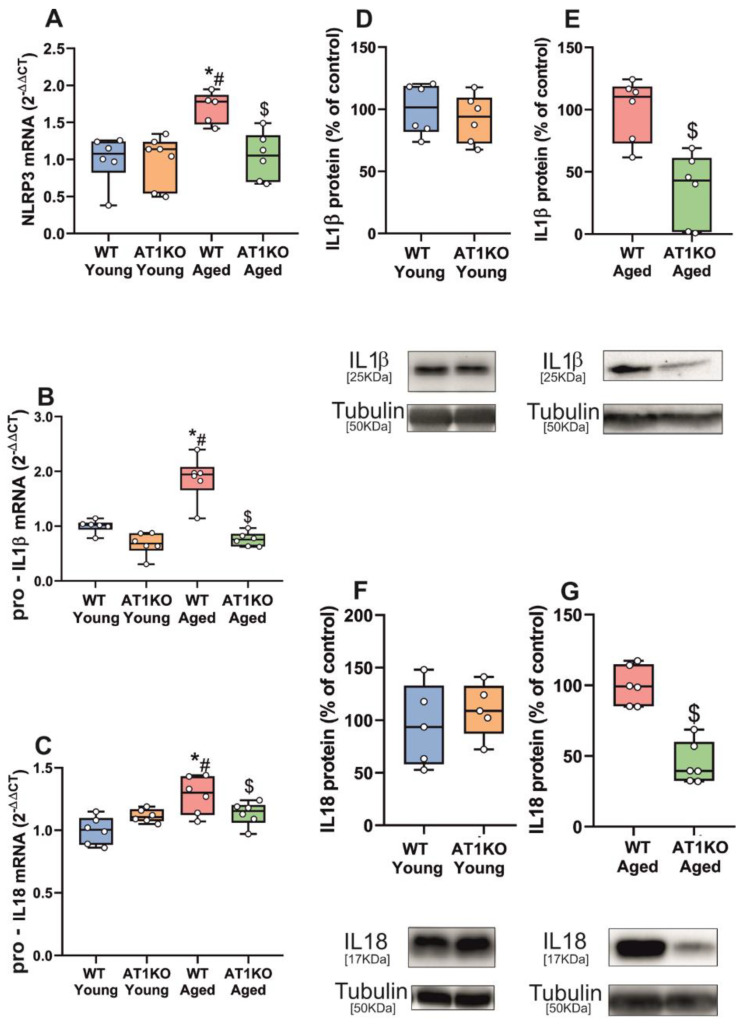
qRT-PCR and WB analysis of NLRP3 inflammasome markers in *substantia nigra* of WT and AT1 KO mice. mRNA expression of NLRP3, pro-IL1β and pro-IL18 inflammasome markers of aged WT mice significantly increased relative to young WT mice (**A**–**C**). mRNA inflammasome markers significantly decreased in aged AT1 KO mice vs. aged WT mice (**A**–**C**). Protein expression of IL1β and IL18 did not show differences in young AT1 KO mice vs. young WT mice (**D**,**F**) and significantly decreased in aged ATI KO mice vs. aged WT mice (**E**,**G**). To determine the relative mRNA expression of each gene of interest vs. the β-Actin housekeeping transcripts, we used the comparative Ct values method (2^−ΔΔCt^). Protein expression was determined relative to the α-tubulin band value and normalized to the control values (100%). Data distribution is shown using box plots with boxes representing the Interquartile range (IQR) and the median and whiskers representing the maximum value (upper whisker) and the minimum (lower whisker). * *p* < 0.05 relative to young WT mice; # *p* < 0.05 relative to young AT1 KO mice, $ *p* < 0.05 relative to aged WT mice.

Consistent with the effects induced by candesartan in young rats, we did not observe a significant change in expression of major NLRP3 inflammasome components (NLRP3, pro-IL1β and pro-IL18) in the young AT1 KO *substantia nigra* relative to that of young WT controls, and aged AT1 KO mice showed significantly less expression of NLRP3, pro-IL1β and pro-IL18 than aged WT mice (Figure 2A–C). We also observed a significant decrease in levels of IL1β and IL18 protein in the *substantia nigra* of aged AT1 KO mice relative to aged WT mice, but not significant differences between young AT1 KO and young WT mice (Figure 2D–G).

**Figure 3:**
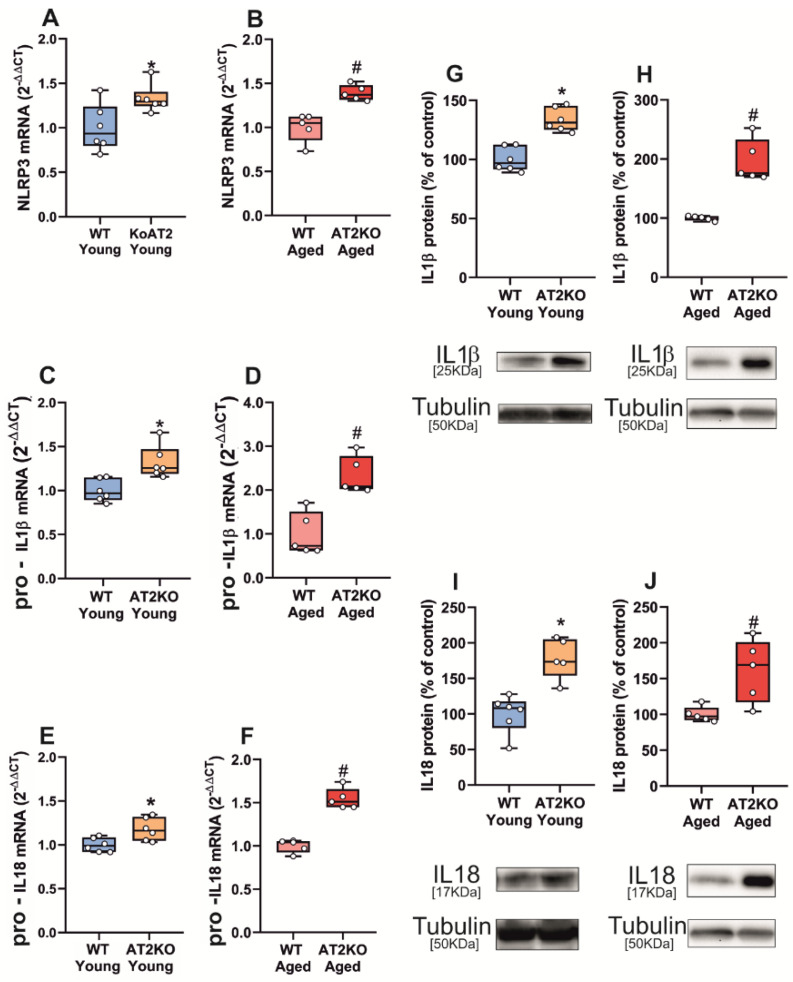
qRT-PCR and WB analysis of NLRP3 inflammasome markers in *substantia nigra* of WT and AT2 KO mice. mRNA expression of NLRP3, pro-IL1β and pro-IL18 inflammasome markers significantly increased in young AT2 KO mice vs. young WT mice (**A**,**C**,**E**) and in aged AT2 KO mice vs. aged WT mice (**B**,**D**,**F**). Protein expression of IL1β and IL18 significantly increased in young AT2 KO mice vs. young WT mice (**G**,**I**) and in aged AT2 KO mice vs. aged WT mice (**H**,**J**). To determine the relative mRNA expression of each gene of interest vs. the β-Actin housekeeping transcripts, we used the comparative Ct values method (2^−ΔΔCt^). Protein expression was determined relative to the α-tubulin band value and normalized to the control values (100%). Data distribution is shown using box plots with boxes representing the Interquartile range (IQR) and the median and whiskers representing the maximum value (upper whisker) and the minimum (lower whisker). * *p* <0.05 relative to young WT mice; # *p* < 0.05 relative to aged WT mice.

In AT2 KO mice, we observed a significant upregulation of mRNA expression for the major NLRP3 inflammasome components (NLRP3, pro-IL1β and pro-IL18) in the *substantia nigra* of young adult AT2 KO, which are known to upregulate AT1 receptors to levels similar to aged WT [36], and also in aged AT2 KO relative to aged WT mice (Figure 3A–F). We also observed a significant increase in levels of IL1β and IL18 protein in the *substantia nigra* of both young and aged AT2 KO mice relative to WT controls (Figure 3G–J).

### 3.2. Up-Regulation of NLRP3 Inflammasome by 6-OHDA-Induced Neurodegeneration Is Mediated by AT1 Receptors

**Figure 4:**
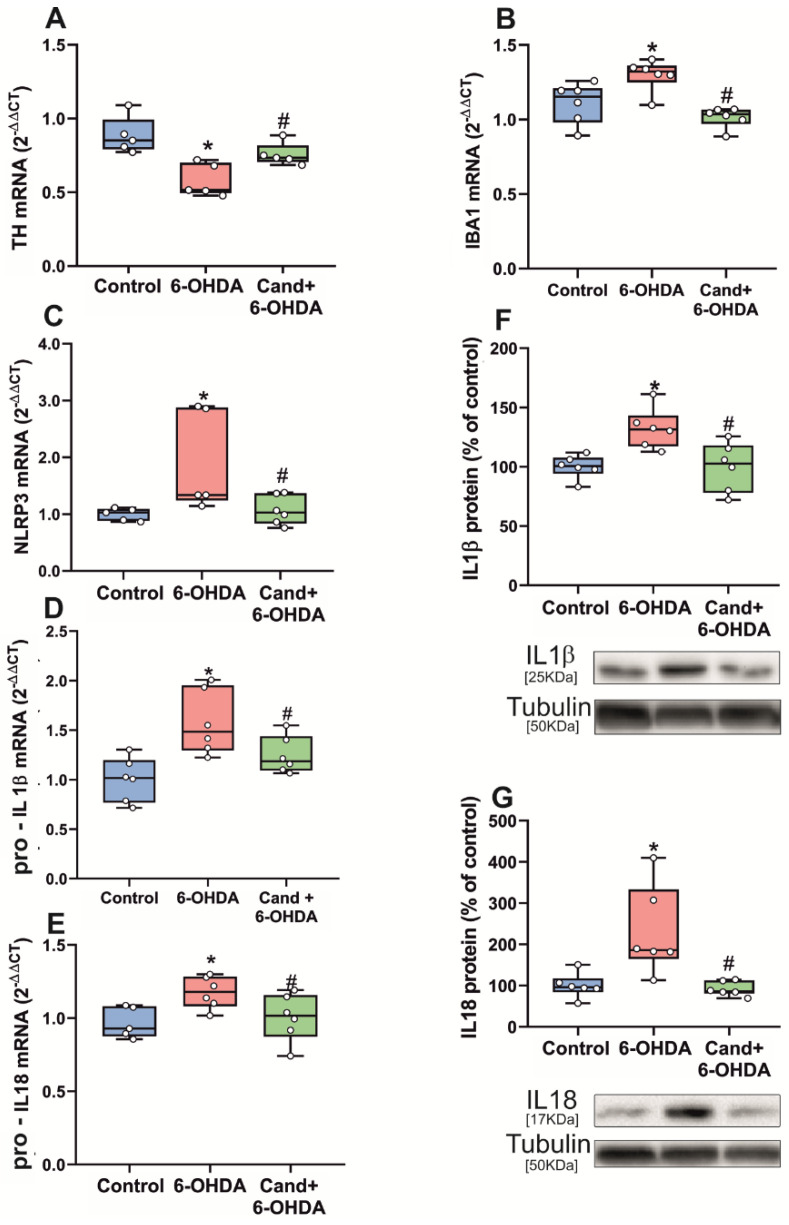
qRT-PCR of TH and IBA1 expression in the *substantia nigra* revealed early effects of intrastriatal 6-hydroxydopamine (6-OHDA) injection on tyrosine hydroxylase (TH) downregulation in dopaminergic neurons and upregulation of the neuroinflammatory microglial response (IBA1) (**A**,**B**). Striatal injection of 6-OHDA significantly increased mRNA expression of NLRP3 inflammasome markers (**C**–**E**) and protein expression of IL1β and IL18 (**F**,**G**) in the *substantia nigra*, which was inhibited by candesartan (**C**–**G**). To determine the relative mRNA expression of each gene of interest vs. the β-Actin housekeeping transcripts, we used the comparative Ct values method (2^−ΔΔCt^). Protein expression was determined relative to the α-tubulin band value and normalized to the control values (100%). Data distribution is shown using box plots with boxes representing the Interquartile range (IQR) and the median and whiskers representing the maximum value (upper whisker) and the minimum (lower whisker). * *p* < 0.05 relative to control rats; # *p* < 0.05 relative to 6-OHDA treated rats.

The *substantia nigra* from rats intrastriatally treated with the dopaminergic neurotoxin 6-OHDA showed a significant decrease in the mRNA expression of TH, reflecting dopaminergic neuron lesion and a significant increase in the mRNA expression of the microglial marker IBA1, indicating the corresponding neuroinflammatory microglial response (Figure 4A,B). As observed in aged rats, these *nigras* with dopaminergic lesion and neuroinflammation showed a significant increase in the expression of mRNA for NLRP3, pro-IL1β and pro-IL18, which was significantly decreased in rats treated with the AT1 antagonist candesartan (Figure 4C–E). Final production of IL1β and IL18 protein was determined by WB, which also showed a significant increase in levels of IL1β and IL18 in the *substantia nigra* of lesioned rats relative to the *substantia nigra* of control rats. As observed above for the step 1 markers, treatment with the AT1 antagonist candesartan significantly reduced the protein levels of IL1β and IL18 (Figure 4F,G).

**Figure 5:**
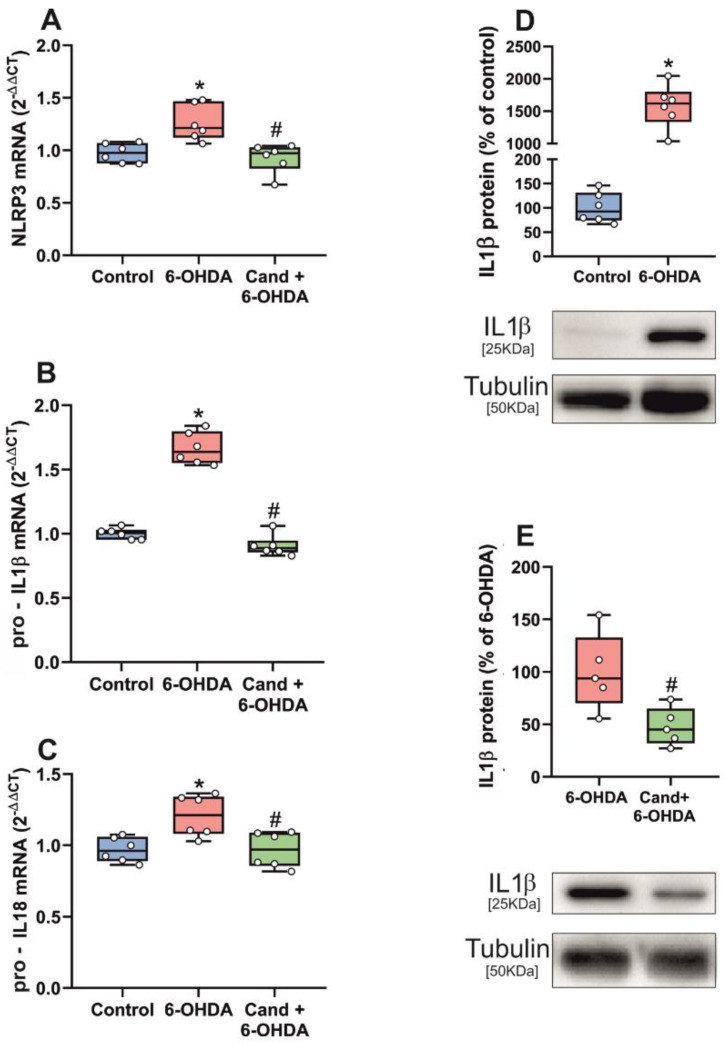
qRT-PCR and WB analysis of expression of NLRP3 inflammasome markers in primary cultures of ventral mesencephalon treated with 6-OHDA. 6-OHDA significantly increased mRNA expression of NLRP3 inflammasome markers (**A**–**C**) and protein expression of IL1β (**D**) in 6-OHDA treated cultures. Candesartan significantly prevented upregulation of the mRNA expression of the inflammasome markers (**A**–**C**) and protein expression of IL1β (**E**). To determine the relative mRNA expression of each gene of interest vs. the β-Actin housekeeping transcripts, we used the comparative Ct values method (2^−ΔΔCt^). Protein expression was determined relative to the α-tubulin band value and normalized to the control values (100%). Data distribution is shown using box plots with boxes representing the Interquartile range (IQR) and the median and whiskers representing the maximum value (upper whisker) and the minimum (lower whisker). * *p* < 0.05 relative to control rats; # *p* < 0.05 relative to 6-OHDA treated rats.

The effects of the dopaminergic lesion were confirmed *in vitro* using primary mesencephalic (neuron-glia) cultures treated with the dopaminergic neurotoxin 6-OHDA. As observed in the *in vivo* model, cultures treated with 6-OHDA showed a significant increase in mRNA expression for NLRP3, pro-IL1β and pro-IL18, which was significantly decreased by simultaneous treatment of cultures with the AT1 antagonist candesartan (Figure 5A–C). Cultures treated with 6-OHDA also showed a significant increase in levels of IL1β and IL18 protein, which were significantly decreased by simultaneous administration of the AT1 antagonist candesartan (Figure 5D,E).

### 3.3. Up-Regulation of NLRP3 Inflammasome by Intraventricular AngII Administration

**Figure 6:**
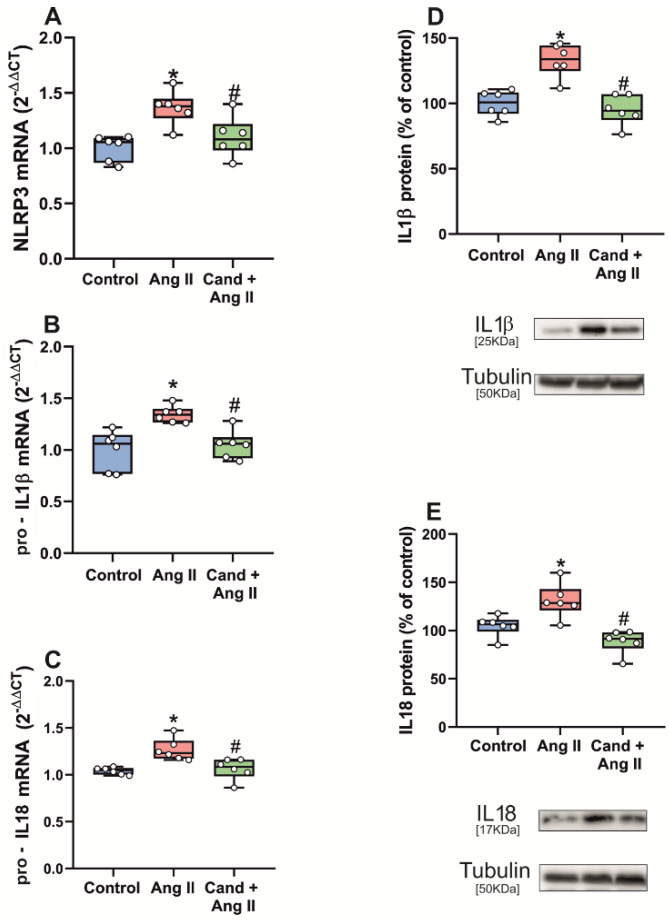
qRT-PCR and WB analysis of expression of NLRP3 inflammasome markers in *substantia nigra* of rats intraventricularly injected with Angiotensin II (AngII). mRNA expression of NLRP3, pro-IL1β and pro-IL18 inflammasome markers (**A**–**C**) and protein expression of IL1β and IL18 (**D**,**E**) significantly increased in the *substantia nigra* vs. non-injected rats. Treatment of rats with candesartan significantly prevented the AngII-induced upregulation of inflammasome markers (**A**–**E**). To determine the relative mRNA expression of each gene of interest vs. the β-Actin housekeeping transcripts, we used the comparative Ct values method (2^−ΔΔCt^). Protein expression was determined relative to the α-tubulin band value and normalized to the control values (100%). Data distribution is shown using box plots with boxes representing the Interquartile range (IQR) and the median and whiskers representing the maximum value (upper whisker) and the minimum (lower whisker). * *p* < 0.05 relative to control rats; # *p* < 0.05 relative to AngII-treated rats.

The enhancing effect of AngII, via AT1 receptors, on inflammasome activation was further confirmed by intraventricular administration of AngII in rats. Administration of AngII induced an increase in the expression of mRNA for major NLRP3 inflammasome components (NLRP3, pro-IL1β and pro-IL18) relative to the expression in the *substantia nigra* of control rats, which was significantly decreased in rats that were treated with the AT1 antagonist candesartan (Figure 6A–C). 

AngII administration also induced a significant increase in levels of step 2 markers IL1β and IL18 in the *substantia nigra* as compared with that of control rats. As indicated above for step 1 markers, treatment of rats with the AT1 antagonist candesartan significantly reduced the effects of AngII administration on protein levels of IL1β and IL18 (Figure 6D,E).

## 4. Discussion

We observed that the *substantia nigra* of aged rats and models of dopaminergic degeneration showed upregulation in transcription of inflammasome-related components (NLRP3, pro-IL1β and pro-IL18; step 1), and IL1β and IL18 protein levels (step 2), and that this was inhibited by treatment with the AT1 receptor antagonist candesartan, showing that activation of the AngII/AT1 axis plays a major role in this process. The role of the AngII/AT1 axis in inflammasome activation in the *substantia nigra* was further confirmed by intraventricular injection of AngII, which induced an increase in expression of inflammasome components (NLRP3, pro-IL1β and pro-IL18), mRNA, and IL1β and IL18 protein levels, which was blocked in rats treated with candesartan.

The role of the prooxidative proinflammatory AT1 receptors in inflammasome activation was further confirmed using young and aged KO mice for AT1 and AT2 AngII receptors. The expression of inflammasome components in young AT1 KO mice was not significantly different to that observed in young healthy controls. However, major inflammasome components (i.e., expression of pro-IL1β and pro-IL18 mRNA, and IL1β and IL18 protein) were significantly upregulated in aged WT mice but not in aged AT1 KO mice. Several previous studies have shown that AT1 receptors are over-expressed in the *substantia nigra* of aged rodents [7,8,31], and particularly in microglial cells [36]. The present observations show that AT1 upregulation in the *nigra* of aged animals leads to upregulation of inflammasome. Interestingly, both young and aged AT2 KO mice showed increased expression of inflammasome components relative to the corresponding WT control mice. This is consistent with the upregulation of AT1 signaling observed both in young and aged AT2 KO mice [36]. Furthermore, this is consistent with our previous observations showing a decrease in AT2 mRNA expression with aging [7,8,36], and that young AT2 KO mice already show characteristics observed in aged WT mice [36].

The present results also show upregulation of inflammasome in early stages of dopaminergic degeneration in the *substantia nigra* and primary mesencephalic cultures, which may trigger the neuroinflammatory process accompanying dopaminergic degeneration, which is known to enhance progression of PD. The results show that AT1 receptors mediate inflammasome activation in models of dopaminergic neuron degeneration.

Several recent studies have shown that physical exercise can inhibit NLRP3 upregulation in several experimental models [37,38,39]. Interestingly, we have previously shown that AT1 receptors and AT1-related inflammatory markers are upregulated in sedentary aged rats and can be decreased by physical exercise [40,41]. The present results suggest that the observed exercise-induced NLRP3 downregulation may be mediated by exercise-induced AT1 downregulation.

A major mechanism induced by activation of the AngII/AT1 axis is production of superoxide derived from NADPH-oxidase complex activation, which generates high levels of superoxide in cells responsible for the inflammatory response, such as microglial cells, and lower levels of superoxide in tissue resident cells such as neurons [42]. Furthermore, a crosstalk between NADPH-oxidase derived superoxide and mitochondria, via mitochondrial ATP-sensitive potassium channels (mitoKATP), leading to further increase in cell ROS levels, has been shown in several tissues including brain *substantia nigra* [14,15]. Interestingly, several studies have shown that NADPH-oxidase- and mitochondrial-derived ROS induce NLRP3 activation by acting particularly on the priming step [43,44], although the responsible mechanism requires further clarification. Consistent with this, neuroprotective properties of NOX2 inhibitors have been related to inhibition of the NLRP3 inflammasome [45]. The present results are also consistent with previous studies in cardiovascular cells [46,47] and brain cardiovascular centers [48] observing that AngII proinflammatory effects are related to activation of NF-κβ nuclear translocation. More recently, we observed that in microglial cells, AngII can induce NF-κβ activation, which is blocked by simultaneous treatment with inhibitors of the NADPH-oxidase complex activation, supporting a major role of NADPH-derived superoxide in the microglial NF-κβ activation in response to AT1 receptor stimulation [13]. This is consistent with the well-known concept that brain NLRP3 is predominantly activated in microglia [22,23]. 

In addition, it was observed that AngII, via AT1 receptors, increases Toll-like receptor (TLR)4-mediated signaling in microglial cells, revealing functional interactions between AT1 receptors and TLR4 for generation of microglial superoxide and microglial activation [49,50], and AT1 receptor blockers reduced TLR4 protein and mRNA expression in human monocytes [51]. The signaling mechanisms underlying the AT1/TLR4 crosstalk remains to be clarified. However, it has been suggested that AT1 activation promotes the transactivation of EGF receptors (EGFRs), and that signaling of EGFRs is necessary for activation of NF- κβ, mediated by TLR4, in response to lipopolysaccharide administration [50,52]. Activation of the NLRP3 inflammasome by TLR4 signaling has been observed in several tissues, including brain [53], and this may contribute to AT1-induced inflammasome activation.

In the present work, we studied interactions of RAS and inflammasome in two closely related models (aging and dopaminergic neurodegeneration). Aging is the most important risk factor for PD. In fact, PD was once considered the result of an accelerated aging [54]. However, it appears that aging itself does not produce significant dopaminergic neuron degeneration but promotes changes leading to higher vulnerability of these neurons to degeneration, and higher risk of PD [55,56]. Normal aging is associated with a prooxidative proinflammatory state that enhances responses to other factors that can induce dopaminergic neuron death [8,57]. 

Dopamine depletion is the hallmark of PD and PD models, and the dopaminergic system is dysregulated during normal aging [55,58]. In the *substantia nigra* of aged rats, we have previously observed a significant decrease of dopamine levels and dopamine type 1 (D1) and type 2 (D2) receptor expression [7,8], which was also observed in different studies in humans and rodents [59,60]. Interestingly, in addition to its broadly described function as a neurotransmitter, dopamine is known to have a role as an immunomodulatory molecule, and dopamine receptors and dopamine are present in immune effector cells [61,62]. It has been observed that dopamine downregulates NLRP3 inflammasome via dopamine D1 receptors and D2 receptors [63,64,65], which is consistent with upregulation of the inflammasome observed in PD models and aged *substantia nigra* in the present experiments, which also show that the brain RAS, and particularly the upregulation of the AngII/AT1 axis mediates this effect.

In previous studies in the *substantia nigra*, we observed that depletion of dopamine (using 6-OHDA or reserpine models) significantly increased the AT1/NADPH-oxidase activity, which was reverted when dopamine levels were restored [66]. Consistent with this, both D1 and D2 KO mice overexpressed AT1 receptors [7]. Previous studies also showed counterregulatory effects between dopamine and AngII in the renal and cardiovascular systems [67,68]. More recently, our group confirmed with *in vitro* experiments [69], that RAS activity is modulated by dopamine and dopamine agonists, both through a decrease of AngII release by astrocytes and through regulation of microglial AngII receptors. Furthermore, dopamine treatment led to a decrease of the microglial AT1/AT2 ratio, consequently inhibiting the prooxidative proinflammatory AT1/NADPH-oxidase/superoxide axis. The present experiments suggest that the proinflammatory effect of AT1 in dopamine-depleted models is mediated via activation of the inflammasome complex.

Given the involvement of neuroinflammation in a considerable number of brain diseases, several promising NLRP3 inhibitors have been investigated. However, the problems of specificity and, particularly, blood–brain barrier (BBB) permeability reduce the possibilities of clinical translation [70]. New promising strategies against these limitations are being developed. However, strategies of repurposing existing drugs that are safe, can cross BBB and can inhibit NLRP3 also constitute a promising avenue for therapy. The present results indicate that AT1 antagonists, widely used in renal and cardiovascular diseases, may be interesting candidates.

## 5. Conclusions

In the *substantia nigra*, the present results support that the prooxidative AngII/AT1 axis of the brain RAS plays an important role in modulating neuroinflammation via upregulation of inflammasome. Inflammasome is upregulated by aging and dopaminergic degeneration, which suggest that the reduction in levels of dopamine observed in early stages of PD and aged brains may enhance neuroinflammation and progression of PD via AngII/AT1 and inflammasome upregulation. The design of BBB permeable orally administrated drugs that target the NLRP3 inflammasome for treatment of PD has had several technical problems up to now. However, AT1 receptor antagonists that cross the BBB, such as candesartan or telmisartan, may play a role as inflammasome inhibitors against aging effects and PD progression.

## Data Availability

The data are available on reasonable request to the corresponding author.
